# Albumin and fibrinogen kinetics in sepsis: a prospective observational study

**DOI:** 10.1186/s13054-021-03860-7

**Published:** 2021-12-17

**Authors:** Keisuke Omiya, Hiroaki Sato, Tamaki Sato, Linda Wykes, Mengyin Hong, Roupen Hatzakorzian, Arnold S. Kristof, Thomas Schricker

**Affiliations:** 1 Department of Anesthesia, McGill University Health Centre Glen Site, Royal Victoria Hospital, 1001 boulevard Decarie, C05-2000, Montreal, QC H4A 3J1 Canada; 2 School of Human Nutrition, McGill University, Montreal, QC Canada; 3 Department of Critical Care Medicine, McGill University Health Centre Glen Site, Royal Victoria Hospital, Montreal, QC Canada; 4 Departments of Critical Care and Medicine, Meakins-Christie Laboratories and Translational Research in Respiratory Diseases Program, Research Institute of the McGill University Health Centre, Montreal, QC Canada

**Keywords:** Albumin, Fibrinogen, Stable isotope tracers, Sepsis

## Abstract

**Background:**

The measurement of circulating substrate concentrations does not provide information about substrate kinetics. It, therefore, remains unclear if a decrease in plasma concentration of albumin, as seen during critical illness, is a consequence of suppressed production in the liver or increased peripheral clearance. In this study, using stable isotope tracer infusions, we measured albumin and fibrinogen kinetics in septic patients and in a control group of non-septic subjects.

**Methods:**

With the approval from the institutional Research Ethics Board and after obtaining written informed consent from patients or their substitute decision maker, mechanically ventilated patients with sepsis and patients scheduled for elective coronary artery bypass grafting were enrolled. Patients in the non-sepsis group were studied on the day before surgery. The stable isotope L-[ring-^2^H_5_]phenylalanine was used to measure absolute synthesis rates (ASR) of albumin and fibrinogen. A priming dose of L-[ring-^2^H_5_]phenylalanine (4 µmol/kg) was given followed by a six-hour infusion at a rate of 0.15 µmol/kg/min. At baseline and hourly thereafter, blood was drawn to measure isotope enrichments by gas chromatography/mass spectrometry. Very low density lipoprotein apolipoprotein-B 100 isotopic enrichment was used to represent the isotopic enrichment of the phenylalanine precursor pool from which the liver synthesizes proteins. Plasma albumin and fibrinogen concentrations were also measured.

**Results:**

Mean plasma albumin in septic patients was decreased when compared to non-septic patients, while synthesis rates were comparable. Mean plasma fibrinogen and ASR in septic patients was increased when compared to non-septic patients. In non-septic patients, no statistically significant correlation between plasma albumin and ASR was observed but plasma fibrinogen significantly correlated with ASR. In septic patients, plasma albumin and fibrinogen significantly correlated with ASR.

**Conclusions:**

While septic patients showed lower plasma albumin levels than non-septic patients, albumin synthesis was similar in the two groups suggesting that hypoalbuminemia during sepsis was not caused by suppressed hepatic production but a result of enhanced clearance from the circulation. Hyperfibrinogenemia in septic patients was a consequence of increased fibrinogen production.

*Trial registration*: ClinicalTrials.gov: NCT02865408 (registered on August 12, 2016) and ClinicalTrials.gov: NCT02549443 (registered on September 15, 2015).

**Supplementary Information:**

The online version contains supplementary material available at 10.1186/s13054-021-03860-7.

## Background

The measurement of the plasma transport protein albumin has been traditionally used to monitor protein and energy deficits in humans. Although plasma albumin concentration may not detect short-term changes in nutritional status, it serves as a consistent marker of chronic protein-calorie malnutrition. The half-life of albumin, the most abundant plasma protein in humans, is 17–19 days [1]. Intravascular albumin reflects approximately 30–40% of the total body albumin, whereas the remainder resides in the extravascular pool [1], i.e., skin, muscle, gut, liver, and subcutaneous tissue [2]. Albumin also plays important regulatory roles in the body’s fluid distribution and acid–base physiology [1, 3, 4]. Hypoalbuminemia can be caused by reduced synthesis, either because of impaired liver function and/or inadequate nutritional protein-energy supply. It also can be the result of enhanced spillover into the extravascular space or excretion via the kidney [5].

Fibrinogen is a so called positive acute phase protein, i.e., its blood levels rise in response to systemic inflammation, tissue injury, and various types of cancer [6, 7]. Elevated levels of fibrinogen have been suggested to be the cause of thrombosis and vascular injury, i.e., complications that are sometimes associated with these conditions [6, 7].

The measurement of circulating substrate concentrations does not provide information about the underlying changes in substrate kinetics. It, therefore, remains unclear if, for example, a decrease in plasma albumin, as seen during critical illness, is a consequence of suppressed production or increased loss. In fact, according to the results of previous studies in patients suffering from inflammation, sepsis, head injury, or burns albumin synthesis rates have been shown to be increased [8–13].

As there are no comprehensive studies on albumin and fibrinogen kinetics in the critically ill, we measured synthesis rates of both proteins in septic patients and in a control group.

## Methods

Data in septic patients were obtained in a (yet unpublished) randomized-controlled trial on the anabolic effects of protein intake during sepsis (ClinicalTrials.gov NCT02865408) which was conducted at the Royal Victoria Hospital (McGill University Health Centre MUHC, Montreal, Quebec, Canada) between March 2017 and June 2019.

Data in non-septic patients are from a randomized-controlled trial on the influence of escalating amino acid amounts on protein balance after open heart surgery (ClinicalTrials.gov NCT02549443). The results of this study, except the albumin kinetics, were published in 2016 [14].

With the approval from the institutional Research Ethics Board and after obtaining written informed consent from patients or their substitute decision maker, mechanically ventilated patients with sepsis (Sepsis Group) and patients scheduled for elective coronary artery bypass grafting (Non-sepsis Group) were enrolled.

Sepsis was defined as an acute change in the total sequential organ failure assessment (SOFA) score ≥ two points secondary to infection [15]. Hence, patients were enrolled during the acute phase of sepsis. Patients in a moribund state, on extracorporeal membrane oxygenation or carbon dioxide removal, on renal replacement therapy, with signs of liver dysfunction, and post organ transplantation were excluded. Clinical scores were calculated at intensive care unit (ICU) admission, including the Acute Physiologic Assessment and Chronic Health Evaluation (APACHE) II was used to assess the severity of illness.

Cardiac patients were studied on the day before surgery, septic patients one day after obtaining consent and enrollment. Patients with severe malnutrition (weight loss > 20% in preceding 3 months, body mass index (BMI) < 20 kg/m^2^), obesity (BMI > 35 kg/m^2^), chronic liver disease (documented chronic viral hepatitis, cirrhosis and abnormal liver function tests), left ventricular ejection fraction < 20%, dialysis and active cancer were excluded.

We used stable isotope tracer kinetics (L-[ring-^2^H_5_]phenylalanine) to measure the absolute synthesis rates (ASR) of albumin and fibrinogen. The ASR of albumin and fibrinogen were determined, following a 12 h fasting period, from 9:00 to 15:00 h, using 6-h-primed-continuous infusions of L-[ring-^2^H_5_]phenylalanine as described previously [16]. A priming dose of L-[ring-^2^H_5_]phenylalanine (4 µmol/kg) was given followed by a six-hour infusion at 0.15 µmol/kg/min. Isotopic enrichment at steady state in the rapidly turning over hepatically-synthesized protein apolipoprotein-B 100 in Very Low Density Lipoprotein (VLDL) was used to represent the isotopic enrichment of the phenylalanine precursor pool from which the liver synthesizes proteins [16, 17]. At baseline, and hourly thereafter, blood was drawn and immediately transferred into pre-chilled tubes containing Na_2_EDTA and a protease inhibitor cocktail (Sigma Aldrich, Oakville, ON, Canada) [18].

To isolate fibrinogen, an ethanol/saline (1:8) solution was added to plasma (4:1) and incubated at 4 °C for 16 h. Samples were then centrifuged at 3000xg for 15 min. The resulting fibrinogen pellet was dissolved in SDS PAGE sample buffer without β-2-mercaptoethanol. The supernatant containing dissolved albumin was mixed 1:1 with SDS PAGE reducing sample buffer. Fibrinogen and albumin were purified separately using 8% SDS PAGE on a MINI-PROTEAN II System (Bio-Rad Laboratories, Hercules, CA, USA). Fibrinogen and albumin samples together with pure standards and molecular weight markers were loaded onto the gel and run at 200 V for 180 and 45 min, respectively. The appropriate protein bands were excised from the gel and hydrolysed in 6 mol/L HCl at 110 °C for 16 h. Amino acids were isolated using cation exchange chromatography (Dowex-50W-X8, Bio Rad Laboratories, Hercules, CA, USA). Phenylalanine was esterified and derivatized to its n-propyl ester heptafluorobutyramide derivative using n-propanol and acetyl chloride, then heptafluorobutyric anhydride. Phenylalanine tracer:tracee ratios were determined by methane negative chemical ionization gas chromatography/mass spectrometry monitoring ions at 383–388*m*/*z*.

Fractional synthesis rates (FSR) of albumin and fibrinogen were calculated using: FSR (%/d) = (*Et*_2_ − *Et*_1_)·24·100/*E*_free_·(*t*_2_ − *t*_1_). *Et*_2_ − *Et*_1_ is the increase in the tracer:tracee ratio of phenylalanine incorporated in the albumin plasma pool using slope of the linear regression line during the final three hours of the infusion (t_2_-t_1_) and E_free_ is the tracer:tracee ratio of liver free phenylalanine at steady state. The ASR of proteins was calculated using: ASR (mg/kg/d) = FSR^.^ conc^.^ PV. Conc is the concentration of the protein in plasma, and PV is the plasma volume calculated by using the hematocrit measured during the infusion period and an average blood volume of 70 mL/kg body weight.

Sample size calculation was based on a previous studies showing a 30 to 50% enhanced albumin ASR in patients with cholecystitis (136 ± 38 mg/kg/d vs. 104 ± 20 mg/kg/d) and burns (111 mg/kg/d vs. 53 mg/kg/d), respectively. Assuming a 40% difference and SD as previously recorded 14 subjects were required in each group to achieve a power level of 80%, with an alpha error of 5%.

Continuous data were analyzed by the unpaired t test or Mann–Whitney *U* test and proportions were analyzed by Fisher’s exact test. The relationship of plasma concentration and synthesis was analyzed by simple regression analysis. Two-sided *P* values < 0.05 were considered statistically significant. All statistical analyses were performed using GraphPad Prism version 8 for Windows (GraphPad Software, San Diego, CA, USA).

## Results

We studied 14 non-septic and 24 septic patients (APACHE score: 21.7 ± 7.8, SOFA score: 8.1 ± 3.5). Details of septic patients are as following: nine patients had complications following open heart surgery, twelve had respiratory problems (seven with pneumonia, three with type 1 respiratory failure, one with type 2 respiratory failure and one with acute respiratory distress syndrome), one patient had substance abuse, one patient suffered from metastatic melanoma, and one patient suffered from aortic thrombosis.

**Fig. 1:**
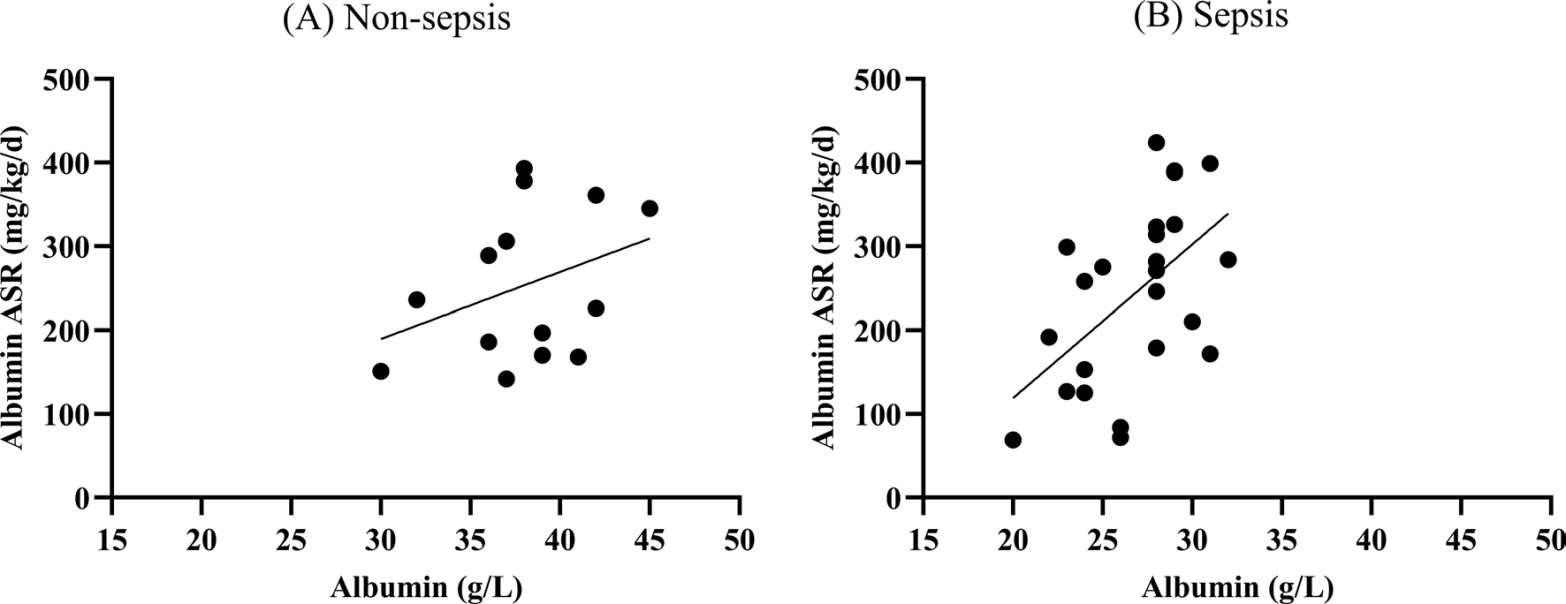
Relationship between albumin (g/L) and albumin ASR (mg/kg/d) in the Non-sepsis and Sepsis group. The relationship was analyzed by simple regression analysis. **A** Non-sepsis group: *Y* = 7.98·*X − *49.8 (*P* = 0.22, *R*^2^ = 0.12), **B** Sepsis group: *Y* = 18.3·*X − *248.0 (*P* = 0.007, *R*^2^ = 0.29). ASR, absolute synthesis rates

**Fig. 2:**
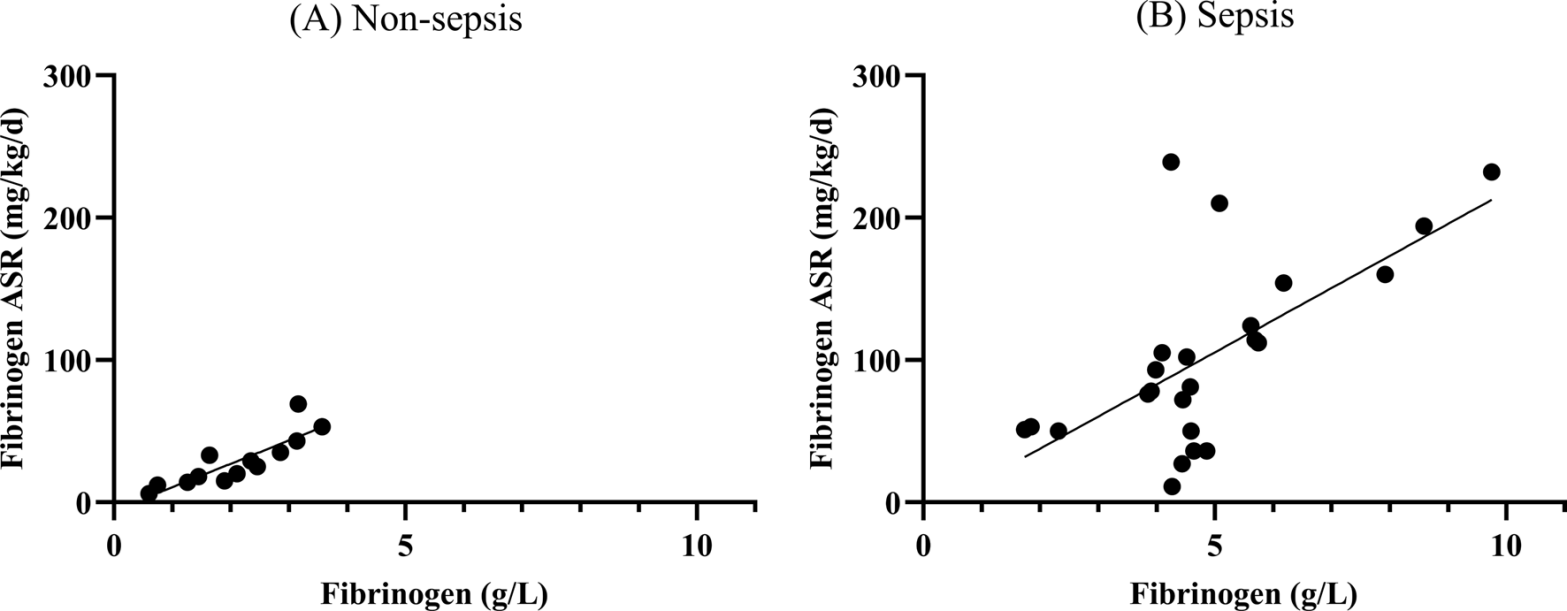
Relationship between plasma fibrinogen (g/L) and fibrinogen ASR (mg/kg/d) in the Non-sepsis and Sepsis group. The relationship was analyzed by simple regression analysis. **A** Non-sepsis group: *Y* = 16.38·*X* − 5.72 (*P* < 0.001, *R*^2^ = 0.73), **B** Sepsis group: *Y* = 22.6·*X* − 7.44 (*P* < 0.001, *R*^2^ = 0.42). ASR, absolute synthesis rates

Patient characteristics are shown in Table 1. Mean plasma albumin concentration in septic patients was lower than in non-septic patients, while synthesis rates were comparable (Table 1). Mean plasma fibrinogen and fibrinogen synthesis rate in septic patients were increased when compared to non-septic patients (Table 1). The ratio of albumin and fibrinogen synthesis rate in septic patients was lower than that in non-septic patients (Table 1). In non-septic patients, no statistically significant correlation between plasma albumin and ASR was observed (Fig. 1A) but plasma fibrinogen significantly correlated with ASR (Fig. 2A). In septic patients, both plasma albumin and fibrinogen significantly correlated with ASR (Figs. 1B, 2B). The original source data sets are available in Additional file 1: Table 1.

**Table 1:** Patient characteristics

	Non-sepsis (*n* = 14)	Sepsis (*n* = 24)	*P* value
Age (y)	62.4 ± 8.8	63.9 ± 12.1	0.69
Female-n (%)	3 (21)	13 (54)	0.09
BMI (kg/m^2^)	27.9 ± 3.8	28.2 ± 5.6	0.86
Diabetes-*n* (%)	2 (14)	7 (29)	0.44
Albumin (g/L)	38.0 ± 3.9	26.8 ± 3.1	< 0.001
Albumin FSR (%/d)	16.2 ± 4.9	18.9 ± 7.8	0.25
Albumin ASR (mg/kg/d)	253.4 ± 89.8	244.3 ± 106.2	0.87
Fibrinogen (g/L)	2.1 ± 0.9	4.9 ± 1.9	< 0.001
Fibrinogen FSR (%/d)	32.4 ± 10.4	44.6 ± 24.5	0.04
Fibrinogen ASR (mg/kg/d)	28.6 ± 18.0	102.5 ± 65.4	< 0.001
Albumin ASR/fibrinogen ASR	12.4 ± 7.6	3.9 ± 5.1	< 0.001

Data are expressed as mean ± standard deviation, *n* (%)

ASR, absolute synthesis rates; BMI, body mass index; FSR, fractional synthesis rates

## Discussion

This is a first report describing the relationship between the circulating concentrations of albumin and fibrinogen and their respective synthesis rates in sepsis. The results of this study indicate that plasma levels of both proteins correlate with their ASR. Hypoalbuminemia during sepsis is most likely a consequence of increased peripheral clearance and not a result of impaired synthesis, while hyperfibrinogenemia is caused by stimulated production. In septic patients, fibrinogen ASR is more than three times higher than in non-septic patients, whereas the rate of albumin synthesis is comparable.

Hypoalbuminemia in the presence of protein malnutrition can be attributed to compromised hepatic synthesis of albumin due to a lack of amino acid availability. In contrast, low albumin levels as seen during cholecystitis [9], sepsis [8] and burns [13] were associated with increased synthesis indicating that, under these conditions, the production of albumin cannot compensate for its enhanced efflux, presumably via capillary leakage into the extravascular space or excretion [2].

In the present protocol, septic patients also were not able to augment albumin production in order to maintain normal albumin plasma levels. The inhibitory effects of increased cortisol [19] and endotoxin levels on albumin synthesis observed in animal models [20] or patients with abdominal abscess [21] may have contributed to this finding. Studies in healthy volunteers [22] showing increased albumin production during the administration of small amounts of endotoxin suggest that the dose of endotoxin may be also metabolically relevant.

By mobilizing polyunsaturated fatty acids from the liver and other tissues [23], and thus enhancing the formation of cytoprotective lipids and free radical suppressors such as lipoxins, resolvins, and protectins, albumin has been shown to exert anti-inflammatory effects with potentially clinical benefits [23]. Considering the importance of the body’s nutritional state for albumin metabolism adequate energy and substrate supply becomes relevant immunologically, particularly in septic patients. While albumin synthesis increased after colorectal cancer surgery with the administration of perioperative hypocaloric nutrition including isonitrogenous amounts of amino acids [16], parenteral amino acid intake did not increase albumin synthesis in septic patients [11].

As illustrated by the results of the present study demonstrating a significant correlation between plasma concentration and synthesis of fibrinogen, hyperfibrinogenemia during sepsis is the result of increased fibrinogen synthesis. The substantially lower albumin ASR to fibrinogen ASR ratio in septic patients indicates a shift in hepatic protein metabolism toward the predominant synthesis of pro-inflammatory proteins further emphasizing the role of fibrinogen as a positive acute phase protein.

Taking into account the pivotal role of this precursor for fibrin gel generation [24], elevated fibrinogen levels may, at least partly, be responsible for coagulopathy and endothelial damage observed during inflammatory states [25, 26]. Conversely, pharmacological inhibition of fibrinolysis, for instance by using tranexamic acid, has been demonstrated to reduce inflammatory responses to open heart surgery and extracorporeal circulation [27, 28].

We acknowledge several limitations of this study.

Firstly, coronary artery disease per se may be associated with inflammatory and metabolic alterations that may have influenced our results. Hence, the control group of subjects scheduled for surgical coronary artery bypass grafting procedures does not represent healthy subjects. Secondly, septic patients typically show interindividual variation of plasma volume [29]. Simple equations as used in the present study to calculate ASR, therefore, may not be entirely accurate for calculating plasma volume in this patient population. Thirdly, albumin clearance was not directly measured. Hence, we cannot exclude the possibility that an altered volume of distribution rather than enhanced spillover into the extravascular space contributed to hypoalbuminemia during sepsis. Fourthly, taking into consideration the potential influence of cortisol and endotoxin on albumin and fibrinogen synthesis, the measurement of both compounds would have allowed better patient characterization and, perhaps, provided information about potential mechanisms underlying the observed changes.

## Conclusion

While septic patients showed lower plasma albumin levels than non-septic patients, albumin synthesis was similar in the two groups suggesting that hypoalbuminemia was not caused by suppressed hepatic production but a consequence of enhanced clearance from the circulation. Hyperfibrinogenemia during sepsis was a result of increased fibrinogen synthesis. Further studies are warranted to investigate whether albumin synthesis can be modified by nutritional support or other pharmacological interventions.

## Supplementary Information

**Additional file 1.** Individual biometric, clinical and metabolic data.

## Data Availability

All data generated or analyzed during this study are included in this published article and its supplementary information files.

## Abbreviations

APACHE: Acute Physiologic Assessment and Chronic Health Evaluation
ASR: Absolute synthesis rates
BMI: Body mass index
FSR: Fractional synthesis rates
ICU: Intensive care unit
PV: Plasma volume
SOFA: Sequential Organ Failure Assessment
VLDL: Very Low Density Lipoprotein
